# Mental Health in Sport (MHS): Improving the Early Intervention Knowledge and Confidence of Elite Sport Staff

**DOI:** 10.3389/fpsyg.2016.00911

**Published:** 2016-06-24

**Authors:** Joshua Sebbens, Peter Hassmén, Dimity Crisp, Kate Wensley

**Affiliations:** ^1^Research Institute for Sport and Exercise, University of CanberraCanberra, ACT, Australia; ^2^Performance Psychology, Australian Institute of SportCanberra, ACT, Australia; ^3^Centre for Applied Psychology, University of CanberraCanberra, ACT, Australia

**Keywords:** athletes, elite sport, early intervention, mental health, mental illness, mental health literacy

## Abstract

Mental illnesses are as prevalent among elite athletes as in the general population. Despite this, there is little research examining how to enhance mental health literacy or helping behaviors in elite sport environments. A Mental Health in Sport (MHS) workshop was therefore developed and its effects on mental health literacy and confidence studied in 166 coaches and support staff working with elite athletes and teams in Australia. Results indicated that participants increased their knowledge of the signs and symptoms of common mental illnesses and were more confident in helping someone who may be experiencing a mental health problem. We conclude that even a very brief intervention can be effective in improving the mental health literacy and confidence of key persons in elite sport environments, and may promote early intervention and timely referral of elite athletes with mental health concerns to appropriate professionals.

## Introduction

During any 1 year, approximately one in five Australians suffer from a common mental illness, and one in four youths aged 16–24 (Australian Bureau of Statistics, 2007). Although the mental health benefits of physical activity have been established (Daley, 2008; Stanton and Reaburn, 2014), elite athletes are not immune to developing a mental illness and are often at the peak of their competitive careers during these high-risk years (Allen and Hopkins, 2015). Moreover, elite athletes experience unique stressors that can have deleterious effects on mental health including sport-related stress (Noblet and Gifford, 2002), injuries (Smith, 1996; Appaneal et al., 2009), living away from home (Bruner et al., 2008), and burnout (Gustafsson et al., 2011). We use the term *mental health problem* in the present study to capture both mental illness (e.g., depression, anxiety) and symptoms of mental illness that may not be severe enough for a diagnosis (Kitchener et al., 2013).

Despite the stressors athletes face, there is a paucity of research on the mental health of elite athletes (Reardon and Factor, 2010; Hughes and Leavey, 2012). A study of elite athletes in Australia reported almost half were experiencing symptoms of a mental health problem, and the proportion meeting caseness cutoffs for mental illness were deemed comparable to community data (Gulliver et al., 2015). More broadly, Rice et al. (2016) conducted a systematic narrative review and also suggested the prevalence of mental illness in elite athletes was comparable to the general population. The authors cautioned that relatively few studies in this area are methodologically rigorous or well reported and that more high-quality systematic and intervention research is required.

There are several barriers to elite athletes accessing help for mental health concerns. Competitive athletes may have less positive attitudes toward help-seeking for mental health problems than non-athletes (Watson, 2005), perhaps partially due to being perceived as a weakness (Bauman, 2016). This perceived stigma among elite athletes is a primary barrier, followed by a lack of awareness of mental health problems, and negative past experiences of seeking help (Gulliver et al., 2012a). Moreover, some sporting organizations may not recognize the prevalence and significance of mental health problems in elite athlete populations (Reardon and Factor, 2010). Access to timely and appropriate care is likely to be restricted if athletes do not feel that the culture of sporting organizations are supportive of these issues (Rice et al., 2016).

One strategy to overcome these barriers to accessing help is to improve the mental health literacy of persons working in elite sport. Mental health literacy has been defined as “knowledge and beliefs about mental disorders which aid their recognition, management or prevention” (Jorm et al., 1997, p. 182). Coaches and support staff in elite sport are currently not required to undergo mental health training and may not possess adequate mental health literacy skills. These frontline staff, however, occupy positions well suited to promote mental health within sport systems due to their established and trusted relationships with athletes (Bapat et al., 2009).

A number of research studies have supported brief interventions as an effective means of improving mental health literacy at an individual level (e.g., Christensen et al., 2004; Deitz et al., 2009; Gulliver et al., 2012b; Dimoff et al., 2016). As well as literacy, an individual's confidence in their agentive capabilities, or self-efficacy, is considered a significant determinant of behavior (e.g., Bandura, 1993). A recent study showed that workplace leaders who received mental health literacy training became more knowledgeable and confident in the promotion of mental health, which in turn resulted in a greater willingness to support colleagues with a mental health problem (Dimoff et al., 2016). Mazzer and Rickwood (2015) found coaches and teachers of young persons were more likely to engage in early intervention behaviors when they perceived themselves as capable, and if they recognized the potential desired outcomes of such behaviors. Given these existing theoretical and empirical links, it is anticipated that knowledge and confidence are important factors that underlie elite coaches' and support staffs' engagement in early intervention behaviors for athlete mental health problems.

Perhaps the most common program currently used to improve mental health literacy and confidence is Mental Health First Aid (MHFA; Kitchener and Jorm, 2002). MHFA is a 2-day program designed to teach members of the public mental health first aid strategies to assist a person developing a mental health problem or in a mental health crisis. There is now a considerable evidence base to suggest MHFA can increase mental health knowledge, increase confidence to help someone with a mental health problem, and thereby increase helping behaviors (Kitchener and Jorm, 2006; Hadlaczky et al., 2014). MHFA is a community-based program that has been deemed efficacious in a variety of settings, including junior and regional sport settings (Bapat et al., 2009; Anderson and Pierce, 2012). The Australian Sports Commission has made access to athlete support a priority in their 10-year plan for elite sport in Australia (Australian Sport Commission, 2012). Improving the mental health literacy of those who spend the most time with athletes (e.g., coaches and support staff) explicitly addresses this priority, and may increase timely referral to appropriate professionals within the sport system.

There are, however, many challenges facing researchers when attempting to deliver and study these existing programs in elite sport. Coaches and support staff lead busy lives. Between training, traveling, and competing, it is increasingly difficult to solicit coaches and support staff to attend time-intensive mental health training programs. Furthermore, existing programs do not address the unique context of elite sport in the etiology and management of mental health problems. Consequently, there exists a need to develop a mental health literacy intervention that is both specific to elite sport and brief enough to encourage uptake by key persons within the system. No research, to our knowledge, has attempted to develop and rigorously evaluate such an intervention.

The purpose of the present study was therefore to evaluate a newly developed Mental Health in Sport (MHS) intervention: a brief applied mental health literacy workshop for coaches and support staff working in elite sport environments. We hypothesized that participants who completed this workshop would increase their mental health literacy as demonstrated by increased knowledge of the signs and symptoms of depression and anxiety, and increased confidence to help someone who may be experiencing a mental health problem.

## Materials and methods

### Participants

Participants (*n* = 166, 22–66 years old) included coaches and trainers, support staff and service providers (e.g., nutritionists, physiotherapists), and managers and administrators working in the Australian high performance sport network. Half of the participants were female (*n* = 83) and the mean age of participants was 37.8 years (*SD* = 10.6). The majority of the sample (*n* = 146, 88%) had a bachelor degree or higher form of tertiary education. Of the participants, 24.1% had some prior mental health training, 30.7% indicated they had personally suffered from a mental health problem at some point in their life, and 53.6% had a family member suffer from a mental health problem.

### Intervention

MHS was created as a 4-h applied workshop designed to educate and up-skill people who work in high performance sport about mental health. The main aim of the workshop was to promote early intervention by equipping participants with the knowledge and confidence to help someone who may be experiencing a mental health problem. Specifically, participants are taught the MHS action plan: *recognize, reach out, refer*, and *remain supportive*. Participants' knowledge was targeted in each of these steps through introducing information and skills in a presentation format. Participants' confidence to help someone experiencing a mental health problem was targeted through applying this knowledge to sport specific videos, case-studies, and role-plays. The MHS action plan was developed to summarize the steps involved in helping someone experiencing a mental health problem. MHS is similar to existing programs (e.g., MHFA) in the focus on early intervention through improving knowledge and confidence, and the use of an action plan framework. MHS differs from existing programs in its brevity, addition of sport specific data and case studies, and the use of role-plays to practice the skills learned. Only anxiety, depression and suicide intervention are covered, and not the effectiveness of various treatments.

In the *recognize* step prevalence rates of anxiety, depression, and suicide in the Australian general population and elite sport were covered, as well as both general and sport specific risk factors for developing a mental illness (e.g., injury, career transitions). The workshop highlighted the signs and symptoms of depression and anxiety, the warning signs that someone may be suicidal, and the impact on individuals, families, and communities. Depression and anxiety were targeted in the content due to high prevalence rates in elite sport (Gulliver et al., 2015; Rice et al., 2016), as is observed in the general population. Other mental illnesses were not addressed due to the time constraints of the workshop. The focus of this step was not on the diagnosis of mental illness, but rather to develop the knowledge and confidence to recognize when someone is struggling.

In the *reach out* step participants were taught how to start a conversation with someone who may be struggling with a mental health problem. The barriers to accessing help for athletes suffering a mental health problem were covered and how to reach out to express concern and offer help. Factors to consider before starting a conversation about mental health were highlighted and participants were instructed in the use of active listening skills to help someone open up and feel understood.

In the *refer* step participants were taught how to refer someone to a mental health professional and who the appropriate professionals are within the sport system. Participants were taught when to and when not to refer, and instances when referral may be necessary against a person's wishes. The reach out and refer steps involved active role-plays where participants practiced the steps of the action plan.

In the *remain supportive* step participants were taught the importance of following up with a person once a referral has been made for a mental health problem. This step was about what to consider after a referral to a professional has been made rather than about early intervention. Participants were taught ways to offer practical and emotional support and that their continued involvement with the person is important during the rehabilitation process.

The role perceptions of the participants were also targeted in the workshop to increase the likelihood of subsequent helping behaviors (Mazzer and Rickwood, 2015). We highlighted the importance of those closest to athletes being mental health advocates and the positive outcomes that may stem from early intervention such as potential performance improvements (Raglin, 2001) and the prevention of acute mental illness that may preclude athletes from training and competition.

The first and fourth authors (registered psychologists working in elite sport environments) co-facilitated the intervention to small groups of 16–32 participants per workshop. The delivery format of MHS across groups was consistent and included lectures, videos, facilitated discussions between participants and presenters, case-studies, and role-plays, and were presented within the context of elite sport. Participants were given an information pack which included business cards of the workshop facilitators, a flyer with a summary of key information from the workshop, a wallet card summarizing the MHS action plan, a suicide information sheet from SANE (“How to help when someone is suicidal,” n.d.^1^), and resources about depression and anxiety from beyondblue (“Anxiety and depression^2^” n.d.; “Anxiety and depression in young people,” n.d.)^3^.

### Design

A randomized controlled trial was not feasible due to workshops being facilitated at several locations around Australia. Thus, a quasi-experimental design was employed whereby workshops were divided into experimental and waitlist comparison groups. Eight workshops in total were administered to participants to assess the efficacy of the intervention. Participants in the first four workshops comprised the experimental group, and participants in the final four workshops comprised the waitlist comparison group.

### Measures

Participants completed online questionnaires prior to attending a workshop (time 1), 2–4 weeks after the experimental group had received the intervention (time 2), and 2–4 weeks after the waitlist comparison group had received the intervention (time 3). The questionnaire contained measures of mental health literacy for depression and anxiety, and confidence to help someone who may be experiencing a mental health problem. Demographic information (i.e., gender, age, and education), mental health history, and prior mental health training were also obtained at time 1 testing.

#### Depression and anxiety literacy

Knowledge of the signs and symptoms of depression and anxiety were measured using 11 items from the Depression Literacy questionnaire (D-lit; Griffiths et al., 2004), and 11 items from the Anxiety Literacy questionnaire (A-lit; Gulliver et al., 2012b) respectively. Only 11 of the original 22 items for each measure were used as the remaining items capture knowledge of effective treatments that were not included in the MHS program. For each statement (e.g., “People with depression may feel guilty when they are not at fault”) participants indicated “True,” “False,” or “I don't know.” Responses were converted to a dichotomous score for each item such that incorrect answers or “I don't know” were scored 0 and correct answers were scored 1. Total scores for the D-lit and A-lit scales ranged from 0 to 11 each, with higher scores indicating greater knowledge. The terms *depression literacy* and *anxiety literacy* are used in the present study for efficiency to reflect knowledge of the signs and symptoms of depression and anxiety respectively. We acknowledge that this is a narrower definition of mental health literacy that includes knowledge of risk factors and evidence-based treatments (Jorm et al., 1997).

#### Confidence

Overall level of confidence in helping someone experiencing a mental health problem was assessed using 4 items developed for the purpose of the study. Participants were asked “How confident are you in: (a) *recognizing* someone with a mental health problem; (b) *reaching out* to someone with a mental health problem; (c) *referring* someone with a mental health problem to a professional; and (d) *supporting* someone with a mental health problem.” Responses were given on a 5-point Likert scale from “not at all” (0), “a little bit” (1), “moderately” (2), “quite a bit” (3), to “very” (4). A principal components analysis with varimax rotation supported a single component extraction that accounted for 72.1% of the variance. Internal consistency among the items was high (α = 0.88); as such mean scores were computed representing a total measure of confidence to help someone who may be experiencing a mental health problem. Scores ranged from 0 to 4, with higher scores indicating greater confidence.

### Procedure

Ethics approval for the study was granted by the University of Canberra's Human Research Ethics Committee (HREC 15-95).

MHS was designed for people working in elite sport with athletes and teams given their existing relationships with, proximity to, and time spent with elite athletes. Therefore, purposive sampling was used to recruit participants. Twelve high performance sport organizations were contacted to ask if they were interested in receiving a MHS workshop for their staff. From these organizations, eight MHS workshops were scheduled in Brisbane, Canberra, Melbourne, and Perth, which included state institutes and academies of sport, national and state sporting organizations, and a sport university. This sample represented a broad spectrum of the high performance sport network in Australia, and included staff working with development pathway athletes through to Olympians and professional athletes. The workshops were then advertised to suitable staff from each organization via email. A total of 202 people expressed interest in attending one of the workshops.

**Figure 1:**
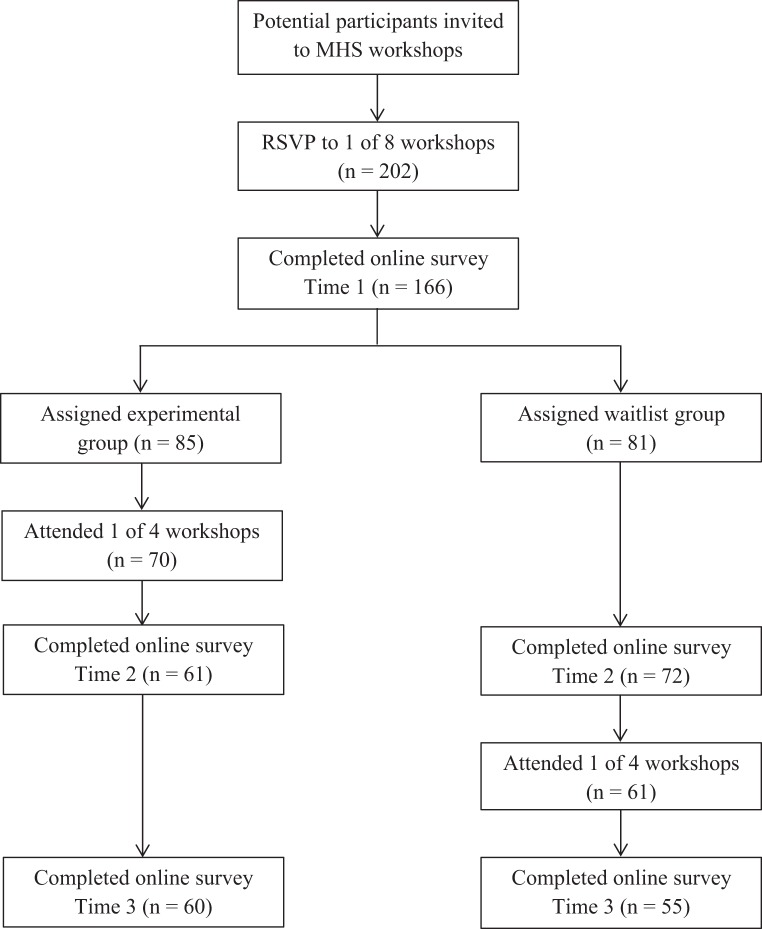
**Flow diagram for participant recruitment and timings of interventions and measurements**.

Participants were emailed details of the research study and invited to complete the baseline survey prior to attending the workshop. One hundred sixty-six respondents consented to participate and completed baseline measurement (time 1). The four workshops comprising the experimental group were then delivered over an 8-day period. Two weeks later all participants were invited to complete the measures again (time 2). The four workshops comprising the waitlist comparison group were then delivered over another 8-day period. Finally, 2 weeks later all participants were invited to complete the measures a third time (time 3). Collectively, at time 1 no participants had received the intervention, at time 2 the experimental group had received the intervention, and at time 3 all groups had received the intervention. The timing of the workshops and measurements were structured to allow both between and within group evaluations of the intervention and to control for history and maturation effects. Figure 1 illustrates the flow of participants through each phase of the study and the timing of the interventions and measurements. All workshop attendees, regardless of study participation, were offered a certificate of completion following each workshop.

### Statistical analyses

Data analysis was conducted using SPSS 21 (IBM SPSS Statistics, Version 21, 2012). Participants received 1 point for each correct item on the D-lit and A-lit, thus missing data for these measures (< 3%) were treated as incorrect answers (Griffiths et al., 2004). There were no missing data for the confidence measure. Longitudinal modeling was used to investigate change over time within a multilevel framework. All models were fitted using an unstructured correlation matrix and by maximum likelihood estimation. Time was defined by fixed time-points representing the intervals of testing (time 1, time 2, time 3). The treatment of time as a categorical variable allowed for comparisons to be made between baseline and subsequent discrete testing points. As there were no significant differences between groups on any of the collected demographic and mental health history data, these variables were not controlled for in the models.

## Results

Baseline (time 1) sample descriptive statistics and dependent variable means by group and time are presented in Table 1. Measures of depression literacy, anxiety literacy, and confidence to help someone who may be struggling with a mental health problem were comparable across experimental and waitlist groups at baseline.

**Table 1:** **Sample descriptive statistics, by sample and intervention group**.

	**T1**	**T2**	**T3**
N	166	133	115
Female, *n* (%)	83 (50.0)		
Age, *M* (S.D.)	37.8 (10.6)		
Bachelor degree or higher, *n* (%)	146 (88.0)		
**GROUP**, ***n*** **(%)**			
Experimental	85 (51.2)	61 (45.9)	60 (52.2)
Waitlist comparison	81 (48.8)	72 (54.1)	55 (47.8)
Depression Literacy, *M* (S.D.), range 0–11	7.94 (1.90)	8.27 (1.86)	8.83 (1.69)
Experimental	7.91 (1.82)	8.95 (1.40)	8.88 (1.62)
Waitlist comparison	7.98 (1.99)	7.69 (2.00)	8.78 (1.78)
Anxiety Literacy, *M* (S.D.), range 0–11	5.53 (2.49)	6.60 (2.47)	7.78 (2.09)
Experimental	5.67 (2.61)	7.70 (1.87)	8.00 (2.12)
Waitlist comparison	5.39 (2.37)	5.67 (2.55)	7.55 (2.05)
Confidence to help, *M* (S.D.), range 0–4	2.15 (0.93)	2.62 (0.88)	3.03 (0.60)
Experimental	2.09 (1.01)	3.11 (0.59)	3.03 (0.61)
Waitlist comparison	2.21 (0.84)	2.20 (0.87)	3.03 (0.60)

### Change in outcomes over time by group

Parameter estimates for the intervention outcomes are presented in Table 2. Although, the magnitude of effects of group, time, and group by time interactions differed for each outcome variable, the pattern of significant effects were consistent. Overall increases in depression literacy, anxiety literacy, and confidence were observed from time 1 to time 3. No significant main effects were found for group on any of the outcome variables; however, significant group by time interactions were indicated for each outcome variable at time 2.

**Table 2:** **Parameter estimates for intervention outcomes**.

	**D-Lit *B* (SE)**	**A-Lit *B* (SE)**	**Confidence *B* (SE)**
Intercept	7.98(0.22)^***^	5.38(0.26)^***^	2.21(0.09)^***^
Experimental Group^a^	−0.07(0.29)	0.29(0.38)	−0.11(0.14)
Time 2b	−0.22(0.18)	0.34(0.20)	0.05(0.05)
Time 3^b^	0.84(0.25)^**^	2.09(0.31)^***^	0.87(0.09)^***^
**INTERVENTION BY TIME INTERACTIONS**			
Experimental Group x Time 2^a^^b^	1.22(0.28)^***^	1.28(0.31)^***^	1.02(0.11)^***^
Experimental Group x Time 3^a^^b^	0.15(0.32)	−0.01(0.42)	0.12(0.13)

a *Reference group is Waitlist group*.

b *Reference time is Time 1*.

*** *P < 0.001*.

** *P < 0.01*.

**Figure 2:**
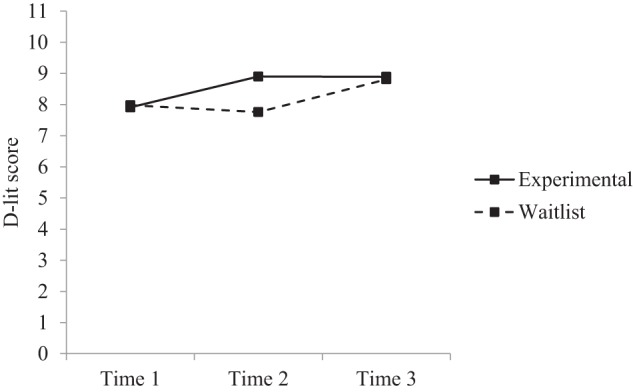
**Estimated marginal means for depression literacy: group × time interaction**.

**Figure 3:**
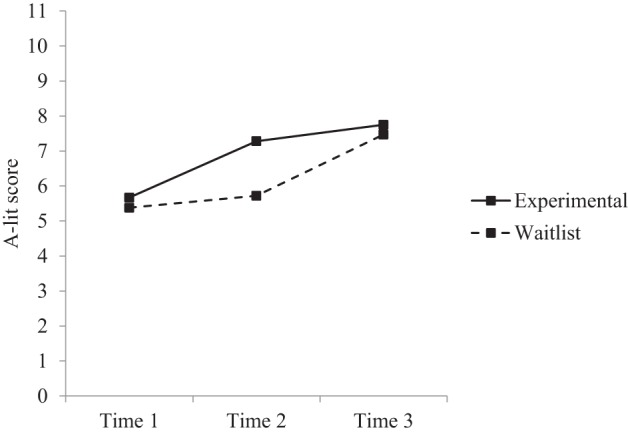
**Estimated marginal means for anxiety literacy: group × time interaction**.

**Figure 4:**
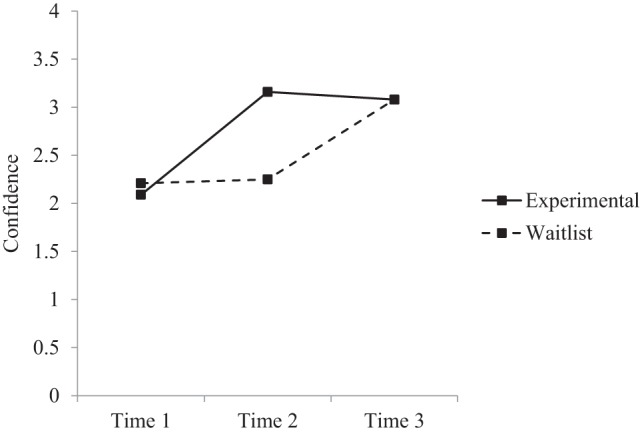
**Estimated marginal means for confidence: group × time interaction**.

*Post-hoc* comparisons were conducted to further examine changes over time across the groups (see Table 3). Results indicated that within the experimental group there were significant increases in depression literacy, anxiety literacy, and confidence from time 1 to time 2, and time 1 to time 3, but no significant differences from time 2 to time 3. Within the waitlist group there were significant increases in depression literacy, anxiety literacy, and confidence from time 1 to time 3, and time 2 to time 3, but no significant differences from time 1 to time 2. Between groups, the experimental group had significantly greater depression literacy, anxiety literacy, and confidence at time 2 compared to the waitlist group. There were no significant differences between groups at time 1 or time 3. Figures 2–4 reflect the group by time interaction for each outcome variable.

**Table 3:** **Pairwise comparisons of estimated marginal means for intervention outcomes**.

	**DLIT MD (SE)**	**ALIT MD (SE)**	**Confidence MD (SE)**
**BETWEEN GROUP CHANGES ACROSS TIME**			
**Waitlist group vs. experimental group**			
Time 1	−0.07(0.29)	0.29(0.38)	−0.11(0.14)
Time 2	1.15(0.29)^**^	1.56(0.38)^**^	0.91(0.13)^***^
Time 3	0.08(0.30)	0.28(0.37)	0.00(0.11)
**WITHIN GROUP CHANGES ACROSS TIME**			
**Experimental group**			
Time 1–Time 2	1.00(0.21)^***^	1.61(0.24)^***^	1.07(0.10)^***^
Time 1–Time 3	0.99(0.20)^***^	2.08(0.29)^***^	0.99(0.10)^***^
Time 2–Time 3	−0.01(0.16)	0.46(0.22)	−0.08(0.06)
**Waitlist group**			
Time 1–Time 2	−0.22(0.18)	0.34(0.20)	0.05(0.05)
Time 1–Time 3	0.84(0.25)^*^	2.09(0.31)^***^	0.87(0.09)^***^
Time 2–Time 3	1.06(0.26)^**^	1.75(0.31)^***^	0.83(0.09)^***^

Bonferroni significance:

*** *P < 0.001*.

** *P < 0.01*.

* *P < 0.05*.

## Discussion

The present study evaluated MHS: a brief mental health literacy workshop designed to promote early intervention for mental health problems in elite sport environments. Using a sample of coaches and support staff working in elite sport, increases in participants' knowledge of the signs and symptoms of depression and anxiety, and confidence to help someone who may be struggling, were observed after attending a MHS workshop and were significantly greater than participants yet to attend. These between and within group effects support our hypotheses.

### Depression and anxiety literacy

The results of the present study suggested that MHS was effective in improving participants' knowledge of the signs and symptoms of depression and anxiety. Prior to attending a workshop (time 1), participants' knowledge of mental illness signs and symptoms was relatively high, reported at 72.2% (*M* = 7.94/11) for depression, and a moderate 50.3% (*M* = 5.53/11) for anxiety across the sample. These data indicated that prior to attending a MHS workshop participants had greater knowledge of depression than they did of anxiety. Although anxiety disorders are more than twice as prevalent as affective disorders in the Australian population (Australian Bureau of Statistics, 2007), the greater knowledge of depression may be explained in part by historical large public campaigns such as beyondblue's *national depression initiative* (Jorm et al., 2005). Levels of depression and anxiety literacy significantly increased for the experimental group following the intervention with significant differences observed between the two groups (time 2). Moreover, these increases were sustained 6–8 weeks after the intervention (time 3). The waitlist group also experienced significant increases in depression and anxiety literacy following their receipt of the intervention (time 3). These increases in depression and anxiety literacy are consistent with previous research that used an internet-based mental health literacy intervention with elite athletes (Gulliver et al., 2012b).

The results further suggested that MHS had a greater effect on knowledge of anxiety than on knowledge of depression. Within the experimental group, anxiety literacy had improved by 18.9% (2.08/11), compared to 9% (0.99/11) for depression at time 3. Similarly, the waitlist group reported increases in anxiety literacy of 19% (2.09/11), and increases in depression literacy of 7.6% (0.84/11) at time 3. Despite the smaller effect on depression literacy, this increase was still statistically significant for both groups. The high level of depression literacy at baseline may have created a ceiling effect and restricted the potential increase to be gained from the brief intervention. In applied terms, identifying approximately one to two additional signs and symptoms of depression and anxiety respectively following the intervention may mean the difference between recognizing an emerging presentation of a mental health problem or not.

### Confidence

The results of the present study supported that MHS was effective in improving participants' confidence to help someone who may be struggling with a mental health problem. Although, not measured herein, increased confidence in agentive capabilities has been linked to an increased likelihood of individuals' engaging in a particular behavior (Bandura, 1993; Vuori et al., 2012). Confidence significantly increased for the experimental group following the intervention with significant differences observed between the two groups (time 2). These increases were sustained 6–8 weeks after the intervention (time 3), which is in line with findings outside of sport (Dimoff et al., 2016). The waitlist group also experienced significant increases in confidence following their receipt of the intervention (time 3). Confidence was moderate (*M* = 2.15/4) across all participants at baseline testing (time 1), and improved to 3.03 out of 4 once all participants had received the intervention (time 3). To provide reference points, a score of 2 on this scale represented “moderately” confident, and a score of 3 represented “quite a bit” confident. This finding is also consistent with MHFA research in junior and regional sport settings (Bapat et al., 2009; Anderson and Pierce, 2012) as well as community settings (Hadlaczky et al., 2014).

### Implications

The main aim of the workshop was to promote early intervention by equipping participants with knowledge and confidence to identify and respond to someone who may be experiencing a mental health problem. Previous research has demonstrated that increased knowledge and confidence following mental health literacy training have been accompanied by more helping behaviors (Kitchener and Jorm, 2006; Hadlaczky et al., 2014). Targeting knowledge and confidence are explicit strategies to increase the likelihood of early intervention, a key behavior-change in the prevention and management of mental illness.

There are several benefits to improving mental health literacy and confidence of coaches and support staff in elite sport environments. First, elite athletes are often within the age groups at greatest risk of developing a mental illness (Australian Bureau of Statistics, 2007; Allen and Hopkins, 2015) and experience a range of unique risk factors within high performance environments (Hughes and Leavey, 2012). Coaches and support staff have close proximity to and established relationships with athletes, therefore occupying positions well suited to be mental health advocates in the daily training environment. Second, 76% of participants in the present study had no prior mental health training. Targeting coaches and support staff is not only important because of the positions they occupy, but because of the current lack of mental health education among this population. Third, research suggests that athletes are reluctant to seek help for mental health problems (Watson, 2005; Gulliver et al., 2012a). MHS empowers coaches and support staff to use their relationships with athletes to intervene early and facilitate help-seeking behaviors. This may prove an effective adjunct strategy to increase instances of early intervention alongside other approaches (e.g., targeting athlete attitudes toward help-seeking directly: Gulliver et al., 2012b). If key persons who work with a high-risk group identify more signs and symptoms of common mental illnesses and also feel more confident to offer help, we believe this represents a significant and important return on investment for a brief intervention.

The knowledge and skills learned by participants in the MHS workshop may be transferrable to contexts outside elite sport. The benefits of MHS may extend beyond helping athletes to helping colleagues, friends, family, and persons in the community. Anecdotal evidence following MHS workshops suggested that some participants have used the skills learned to make contact with friends and family members they were concerned about. Some participants indicated that they have recognized signs and symptoms in themselves and have been prompted to take action on their own mental health. Further research is needed to explore these additional potential benefits of the MHS workshop.

The present study also offers preliminary evidence that brief and context specific workshops may be a viable alternative to multi-day generic mental health literacy programs (e.g., MHFA; Kitchener and Jorm, 2002). Although, we would defer to MHFA as the gold standard in public mental health literacy training, the content and structure of this program may not be optimal in all environments. We have found it increasingly difficult to reach coaches and support staff using such programs in elite sport environments. Developing brief alternatives that encourage uptake by key persons and that incorporate research, experience, and case examples that are context specific is one strategy to increase the reach and relevance of mental health literacy training across different environments. Further research is needed to evaluate the efficacy of brief mental health literacy workshops over both the short and long-term.

### Limitations and future directions

The results of the present study should be interpreted in light of several limitations. First, a randomized controlled trial was not feasible so a quasi-experimental design was adopted. There may be differences in unobserved and potentially confounding variables between groups due to lack of random assignment, despite the waitlist and experimental groups being comparable on observed variables at baseline testing. The chosen design, however, was deemed more robust than a single group pre-post intervention design. Second, MHS only focuses on the signs and symptoms of two mental illnesses (depression and anxiety) and a brief suicide intervention protocol. Knowledge of signs and symptoms of common mental illnesses is a necessarily narrower definition of mental health literacy than is used in programs that teach participants about a greater range of disorders and the effectiveness of different treatments (e.g., MHFA). The high scores for depression literacy at baseline may be explained, in part, due to this narrower definition. This focus, however, was necessary given the time constraints of a brief intervention, and was deemed the best strategy to improve engagement in mental health education and increase instances of early intervention. Finally, the present study did not assess actual behavior change following the workshop. Although greater mental health knowledge and confidence may be accompanied by an increase in helping behaviors (Bandura, 1993; Kitchener and Jorm, 2006; Hadlaczky et al., 2014; Dimoff et al., 2016) this association requires further investigation.

Future research should attempt to assess the longer-term impact of brief interventions on mental health literacy, levels of confidence, and behavior change. Longitudinal designs could track changes in referrals for mental health problems within elite sport environments, or qualitative methods could be employed to examine if and how participants use the skills learned in the workshop. Other outcome variables that may predict helping behaviors should also be explored, such as coaches' and support staff's role perceptions relating early intervention for mental health problems (Mazzer and Rickwood, 2015). Moreover, athletes' support systems involve not only coaches and support staff, but also their friends and families (Gulliver et al., 2012a). Future research may examine MHS as a workshop to offer other people who are in positions to recognize and respond to emerging mental health problems in athletes. Finally, evaluating MHS's effect on stigma may also be an interesting line of enquiry given such attitudes may currently undermine early intervention efforts in elite sport (Gulliver et al., 2012a).

To our knowledge this is the first study to develop and evaluate a brief mental health literacy workshop specific to elite sport environments. Improving coaches' and support staff's mental health knowledge and confidence to help others may improve early identification and timely referral of mental health problems in elite athletes. The present study supports MHS as an effective alternative to generic mental health literacy workshops for use in elite sport environments. Further research is required to examine the longer-term benefits and applicability of the workshop.

## Author contributions

JS developed the study concept. KW developed the pilot version of the MHS workshop and JS and KW developed the final version. All authors contributed to the study design. Data collection was performed by JS and was analysed under the supervision of DC. JS drafted the manuscript. All authors contributed to the editing process.

### Conflict of interest statement

The authors declare that the research was conducted in the absence of any commercial or financial relationships that could be construed as a potential conflict of interest.

## Footnotes

^1^How to Help When Someone is Suicidal: Factsheet & Guide. (n.d.). Retrieved June 30, 2015, from https://www.sane.org/mental-health-and-illness/facts-and-guides/sane-steps-how-to-help-when-someone-is-suicidal

^2^Anxiety and Depression: An Information Booklet. (n.d.). Retrieved June 30, 2015, from http://resources.beyondblue.org.au/prism/file?token=BL/0885

^3^Anxiety and Depression in Young People: What You Need to Know. (n.d.). Retrieved June 30, 2015, from http://resources.beyondblue.org.au/prism/file?token=BL/1060
